# The Ketogenic Diet in the Neonatal Intensive Care Setting: The Case of a Preterm Newborn With Mitochondrial DNA Depletion Syndrome Type 13 (MTDPS13)

**DOI:** 10.1155/crig/6492770

**Published:** 2026-02-02

**Authors:** Gabriele D’Amato, Mattia Gentile, Rossella Carella, Antonio Giannini, Maria Felicia Faienza, Albina Tummolo

**Affiliations:** ^1^ Neonatal Intensive Care Unit, Di Venere Hospital, Bari, 70012, Italy; ^2^ UOC Laboratorio di Genetica Medica, PO Di Venere-ASL Bari, Bari, 70012, Italy; ^3^ Department of Metabolic Diseases and Clinical Genetics, Children Hospital Giovanni XXIII, Bari, Italy; ^4^ UOC Radiodiagnostica, P.O. Di Venere, Bari, Italy; ^5^ Pediatric Unit, Department of Precision and Regenerative Medicine and Ionian Area, University of Bari “Aldo Moro”, Bari, 70124, Italy, uniba.it

## Abstract

**Background:**

Mitochondrial DNA depletion syndrome 13 (MTDPS13) is an autosomal recessive disorder presenting in early infancy with encephalopathy, hypotonia, lactic acidosis, and severe global developmental delay. Patient‐derived cells typically exhibit impaired mitochondrial oxidative phosphorylation and a marked reduction in mitochondrial DNA (mtDNA) copy number.

**Case Report:**

We report the case of a male preterm neonate born at 31 + 3 weeks of gestation following a pregnancy marked by severe polyhydramnios. At birth, his weight was 1400 g. Physical examination revealed dysmorphic features, redundant and lax skin, and generalized muscular hypotonia. Laboratory investigations showed marked lactic acidosis associated with lactic aciduria, ketonuria, and urinary biomarkers indicating activation of preoxidative phosphorylation biochemical pathways to sustain ATP production. Echocardiography demonstrated mild, early‐onset hypertrophic cardiomyopathy.

**The Exome Analysis Clinical and Biochemical Markers:** The exome analysis, performed within the first week of life, highlighted a pathogenic variant in homozygous state of *FBXL4* gene (c.1648_1649delGA), which led to the diagnosis of MTDPS13. In this clinical contest, a ketogenic diet (KD) was started with a daily caloric intake of 120 kcal/kg and an initial ketogenic ratio of 1:1. These intakes were administered both with a parenteral nutrition and continuous nasogastric tube feeding and were gradually increased and adapted on a day‐by‐day basis according to lactic acidosis, growth increase, and common metabolic parameters such as glucose, electrolytes, creatinine, and blood urea nitrogen. After 3 days of this treatment approach, a significant reduction in lactate levels and improvement in acid–base balance and growth trend were observed along with clinical and cardiovascular parameters. At discharge from neonatal intensive care unit, the KD was continued at home and during follow‐up. The infant showed stability in the clinical and biochemical markers.

**Conclusions:**

This is the first documented report of the use of a KD in a preterm neonate with this mitochondrial disorder during the early days of life. Prompt genetic confirmation and early initiation of KD may enable a more targeted and effective management of MTDPS within the neonatal intensive care setting.

## 1. Introduction

Mitochondria form a dynamic population of organelles in all eukaryotic cells, primarily responsible for ATP generation through the electron transport chain and oxidative phosphorylation. Beyond energy production, they participate in heat generation, apoptosis, regulation of reactive oxygen species (ROS), intracellular Ca^2+^ homeostasis, steroid and heme biosynthesis, and lipid metabolism [1]. Mitochondrial quality and quantity are preserved through mitophagy, a selective form of autophagy activated by conditions such as hypoxia, cellular differentiation, or mitochondrial damage. The relevance of maintaining an adequate mitochondrial DNA (mtDNA) copy number is underscored by mitochondrial DNA depletion syndromes (MTDPSs), a group of disorders characterized by significantly reduced mtDNA levels in affected tissues [2, 3]. These syndromes result from pathogenic variants in nuclear genes involved in mtDNA replication, nucleotide pool balance, and mitochondrial dynamics. Among the nuclear‐encoded proteins required for mtDNA maintenance, *FBXL4* (F‐box and leucine‐rich repeat protein 4; MIM #605654) plays a crucial role [4, 5]. Since its initial description in 2013, 36 pathogenic *FBXL4* variants have been identified in approximately 100 individuals. No specific therapy is currently available, and management remains supportive, addressing nutritional needs and neurological complications such as developmental delay, seizures, cardiac dysfunction, ophthalmologic involvement, and hearing loss [6, 7].

Here, we report the case of a male preterm neonate with mitochondrial DNA depletion syndrome 13 (MTDPS13) who was successfully managed in the neonatal intensive care unit (NICU) using a ketogenic diet (KD) approach.

## 2. Case Report

A male preterm neonate was born at 31 + 3 weeks of gestational age (GA) by emergency caesarean section due to severe polyhydramnios. His birth weight was 1400 g (−0.5 SDS), length was 40 cm (−0.4 SDS), head circumference was 30 cm (+0.7 SDS), and his APGAR scores at 1 and 5 min were normal (8 and 10, respectively). In the NICU, he required noninvasive ventilation via nasal prongs for the first 4 days, without supplemental oxygen. He presented with a progeroid facial appearance, redundant and lax skin, diffuse muscular hypotrophy, and syndactyly of the 2nd and 3rd toes on the left foot. Additional dysmorphic features included a smooth and elongated philtrum, macrofontanelle, widened nasal root and bridge, and low‐set, posteriorly rotated ears. Neurological examination revealed marked and generalized hypotonia.

Arterial blood gas analysis showed moderate metabolic acidosis with elevated lactate (16 mmol/L). After secondary causes of metabolic acidosis were excluded, the condition persisted despite intravenous sodium bicarbonate administration. Continuous sodium bicarbonate infusion was therefore initiated, together with a reduction in protein intake (1 g/kg/day) within total parenteral nutrition (TPN), pending results from newborn screening as well as plasma amino acid profile, serum ammonia, and urinary organic acids to investigate a suspected inborn error of metabolism (IEM).

The results of the extended newborn screening excluded an organic acidemia. Plasma ammonia level was in the normal range (45 micromol/L, normal value: 71 ± 26).

Nevertheless, the urinary organic acid profile was abnormal and showed lactic, pyruvic, and 2‐hydroxybutyric aciduria, together with ketonuria and elevated levels of several Krebs cycle intermediates, including malic, succinic, fumaric, and 2‐ketoglutaric acids. The plasma amino acid profile revealed increased concentrations of alanine, proline, isoleucine, leucine, and lysine. Taken together, these findings raised suspicion of a mitochondrial disorder.

### 2.1. Molecular Analysis

Whole‐exome sequencing performed on the fifth day of life identified a homozygous variant in the *FBXL4* gene (NM_001278716.2): c.1648_1649delGA, located at position chr 6:99323344‐99323345 (hg19, Exon 9). This frameshift variant results in a premature stop codon and is predicted to produce a truncated protein (p.Asp550HisfsTer2). The variant is reported in ClinVar (RCV000502558) as pathogenic and is consistently classified as disease‐causing according to the American College of Medical Genetics and Genomics (ACMG) criteria and associated in previous cases with severe clinical course and early death [4].

### 2.2. Nutritional Management

On the first day of life, enteral nutrition was initiated with maternal breast milk. In accordance with the European Society for Clinical Nutrition and Metabolism (ESPEN) guidelines [8], a volume of 1 mL/h (24 mL/day, corresponding to approximately 17 mL/kg/day) was planned, in combination with TPN providing a relatively low protein intake for a preterm neonate. At that time, his body weight was 1250 g, and a ketogenic‐inspired macronutrient regimen was prescribed due to the suspected mitochondrial disorder. Protein intake was set at 3 g/kg/day (as recommended for preterm infants) [9], while carbohydrate (5 g/kg/day) and lipid (7 g/kg/day) intakes were adjusted to achieve a ketogenic ratio of 0.9:1, providing a total energy intake of 120 kcal/day (97 kcal/kg/day).

Concurrently, high‐dose vitamin supplementation was initiated, including coenzyme Q10 (15 mg/kg/day), biotin (10 mg/day), thiamine (300 mg/day), and riboflavin (300 mg/day). On Day 5, enteral feeding via gavage was started using a ketogenic mixture with a 0.9:1 ratio, prepared by combining a ketogenic formula (3:1 ratio) with a preterm infant–specific formula (Table 1). The infusion was initiated at 1.5 mL/h, corresponding to a total volume of 30 mL/kg/day and providing 30 kcal/day (24 kcal/kg/day). When added to the 120 kcal/day provided by parenteral nutrition, the total daily energy intake reached 150 kcal (120 kcal/kg/day), consistent with the estimated energy requirements for a preterm infant weighing 1.25 kg.

**TABLE 1:** Bromatological composition of diet over time (both parenteral and enteral intakes).

**Weight (g)**	**Day 1**		**Day 3**		**Day 5**		**Day 6**		**Day 7**		**Day 8**		**Day 12**		**Day 50**	**Day 71**
	**1400**		**1250**										**1500**		**2300**	**2715**
	**EN**	**PN**	**EN**	**PN**	**EN**	**PN**	**EN**	**PN**	**EN**	**PN**	**EN**	**PN**	**EN**	**PN**	**OF**	**OF**
	**Human milk**	**Glucose infusion**		**Ketogenic infusion**	**Ketogenic mixture**	**Ketogenic infusion**	**Ketogenic mixture**	**Ketogenic infusion**	**Ketogenic mixture**	**Ketogenic infusion**	**Ketogenic mixture**		**Ketogenic mixture**		**Ketogenic mixture**	**Ketogenic mixture**

Volume/day (mL)	24	n.a	n.a	n.a	37.5	n.a	72	n.a	112.5	n.a	150	n.a	220	n.a	360	400
kcal/day	16.2	n.a	n.a	120	30	120	60	90	90	60	120	n.a	277	n.a	350	414
kcal/kg/day	12.96	n.a	n.a	96	24	96	48	72	72	48	96	n.a	185	n.a	152	152
Proteins g/day	0.3	n.a	n.a	3.75	0.6	3.75	1.2	2.8	1.8	1.8	2.4	n.a	5.3	n.a	6.6	10.4
Carbohydrates g/day	1.5	n.a	n.a	6.25	1.7	6.25	3.4	4.7	5.1	3.1	6.8	n.a	16	n.a	20.2	20.8
Lipids g/day	0.8	n.a	n.a	8.75	2.16	8.75	4.32	6.6	6.48	4.4	8.64	n.a	21.3	n.a	26.8	31.8
Ketogenic ratio	n.a	n.a	n.a	0.9:1	0.9:1	0.9:1	0.9:1	0.9:1	0.9:1	0.9:1	0.9:1	n.a	1:1	n.a	1:1	1:1

Abbreviations: EN, enteral nutrition; n.a, not available; OF, oral feeding; PN, parenteral nutrition.

On the 6th day of life, given the satisfactory tolerance, the enteral feeding volume was increased to 60 mL/kg/day, with the infusion rate adjusted to 3 mL/h. In parallel, parenteral nutrition was progressively reduced to 90 kcal/day to maintain the total energy intake and daily fluid volume within the optimal target range. On the following day, the enteral intake reached 90 mL/kg/day, corresponding to an infusion rate of 4.5 mL/h. Thereafter, daily increases of approximately 30 mL/kg/day were implemented, and by Day 8 of life (i.e., 5 days after initiation of the KD), a total enteral volume of 150 mL/day (120 mL/kg/day) was achieved, providing 120 kcal/kg/day.

**FIGURE 1:**
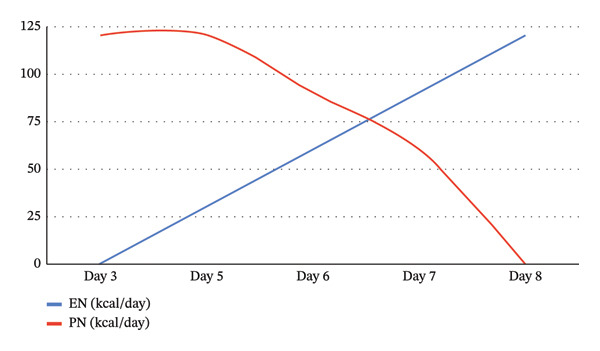
Caloric intake over time: gradual shift from parental to enteral support. EN: enteral nutrition; PN: parenteral nutrition.

Figure 1 shows the contribution of parenteral and enteral nutrition to the total caloric intake over the first 8 days of life. The progressive increase in the enteral feeding infusion rate was carried out in accordance with the European Society for Paediatric Gastroenterology Hepatology and Nutrition (ESPGHAN) guidelines for the transition from minimal enteral feeding (MEF) to full enteral feeding in preterm infants within the neonatal intensive care setting [9].

The nutritional regimen was well tolerated and provided an energy intake sufficient to achieve full enteral feeding, in line with ESPEN recommendations, over the subsequent days until Day 12 of postnatal life. By that time, a weight gain of 250 g had been achieved, corresponding to an average daily increase of approximately 35 g/day. The energy intake was subsequently increased to 277 kcal/day (185 kcal/kg/day) while maintaining a ketogenic ratio of 1:1. During this phase, a small portion of the ketogenic mixture was administered orally to facilitate a gradual transition to oral feeding [9, 10]. In the following days, the diet was progressively adjusted to meet the infant’s nutritional requirements in relation to weight gain, while maintaining a 1:1 ketogenic ratio.

### 2.3. Clinical and Instrumental Features

Following the initiation of the KD together with multivitamin supplementation, a gradual resolution of lactic acidosis and lactate overproduction was observed (lactate 3.5 mmol/L, base excess −1.5 mmol/L, and HCO_3_^−^ 22.6 mmol/L). Sodium bicarbonate infusion was discontinued on the 4th day of life.

During the NICU stay, a brain ultrasound performed on the 2nd day of life revealed mild enlargement of the lateral ventricles and diffusely hyperechogenic periventricular white matter with extension into the subcortical regions. In addition, a single small echolucent white matter cyst, measuring 3 mm and located near the outer corner of the right frontal horn of the lateral ventricle, was identified. Transthoracic echocardiography revealed mild biventricular myocardial hypertrophy with normal functional parameters on Doppler assessment. The clinical course was notable for steady weight gain, poor sucking, ongoing requirement for tube feeding, and reduced muscle tone with limited spontaneous movements. A follow‐up brain ultrasound performed at term‐equivalent age demonstrated moderate enlargement of the ventricular system, persistently hyperechogenic white matter, thinning of the corpus callosum, and hypoplasia of the cerebellar vermis and left cerebellar hemisphere.

**FIGURE 2:**
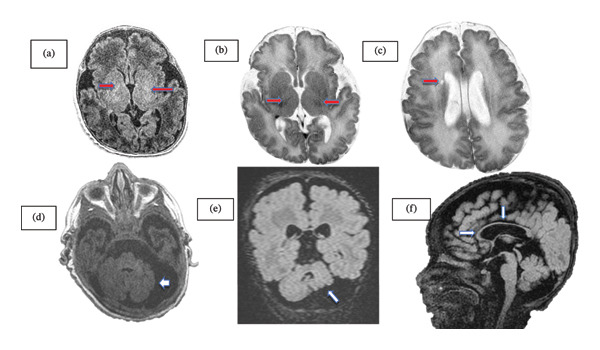
Brain MRI images. (a) Volumetric T1‐weighted 3D BRAVO (brain of volume) FSPGR (inversion recovery fast spoiled gradient echo) and (b) T2‐weighted FRFSE (fast recovery fast spin echo) sequence axial images at level of basal ganglia: mild delayed myelination (red arrows). (c) T2‐weighted FRFSE sequence axial image at the level of caudate head nuclei: small fluid cyst of the periventricular frontal horn of the right lateral cerebral ventricle (red arrow). (d) T1‐weighted M3D BRAVO (brain of volume) FSPGR (fast spoiled gradient echo) and (e) 3D (volumetric) cube (isotropic voxel) T2‐weighted fluid‐attenuated inversion recovery (FLAIR) axial and coronal sequences, respectively, of posterior cerebral fossa: high‐grade cerebellar hypoplasia (white arrows). (f) 3D (volumetric) cube (isotropic voxel) T2‐weighted FLAIR sagittal image: thin corpus callosum (white arrows). ^∗^All the images are obtained with MRI Scanner General Electric Signa Horizon, 1.5 T HDxT.

One week later, a brain magnetic resonance imaging (MRI) was performed, which revealed morphologic, diffusion, signal, and contrast‐enhancement abnormalities consistent with bilateral cerebellar hemispheric hypoplasia, delayed white‐matter myelination, a small cerebrospinal fluid (CSF)–like cyst adjacent to the frontal horn of the right lateral ventricle, and hypogenesis of the corpus callosum (Figure 2). The infant gradually showed improvement in sucking and oral feeding abilities around 39 weeks of post‐conceptional age. Before discharge, auditory brainstem response (ABR) testing demonstrated absent bilateral responses. Ophthalmologic examination revealed a pale optic nerve and hyperpigmentation in the macula.

The neonate was discharged home at 41 + 4 weeks of post‐conceptional age with a weight of 2715 g. In the final days of hospitalization, good tolerance to the prescribed feedings was confirmed, and full oral feeding was achieved. Given the infant’s limited ability to further increase caloric intake and feeding volume, the established nutritional regimen was maintained during the immediate postdischarge period.

### 2.4. Management After Discharge

At home, nutritional requirements were met using a mixture composed of a ketogenic formula at a 3:1 ratio, water, and preterm infant formula, combined to maintain a ketogenic regimen with a final ratio of 1:1. The mixture was administered orally in eight feeds per day, each with a volume of 50 mL, providing a total daily energy intake of 414 kcal, corresponding to 152 kcal/kg/day.

At the clinical assessment performed 10 days after discharge, the newborn exhibited a strong cry and was able to turn and respond to sounds by blinking. His weight gain at home averaged approximately 25 g/day.

At 4 months of age, he was admitted for respiratory distress, tachycardia, severe lactic acidosis, increased inflammation indices, and food refusal. The diagnosis sepsis was made with gradual worsening of cardiac function and exitus at 6 months of age.

## 3. Discussion

We report the case of a preterm neonate with MTDPS13 who showed a remarkable clinical response to KD therapy, including normalization of metabolic acidosis. Although multiple pathogenic variants in *FBXL4* have been identified as causative of MTDPS13, only a few cases of FBXL4 deficiency treated with a KD have been reported in the literature, and none during the neonatal period. In a previous report, a 4‐year‐old girl with a pathogenic variant in the *FBXL4* gene was initially started on standard parenteral nutrition, which was gradually transitioned to parenteral KD; her lactate levels decreased following the initiation of parenteral KD, and she was subsequently discharged on enteral KD [11]. In another study, KD was administered to an 8‐month‐old boy with FBXL4 deficiency for 5 days, although details regarding the composition of the KD, mode of administration, and clinical, laboratory, or instrumental outcomes were not reported [12].

The KD, typically characterized by high lipid, variable protein, and low carbohydrate content, has been used in the treatment of intractable epilepsy since the 1920s [13]. Ketone bodies serve as precursors for the synthesis of fats and amino acids and stabilize neuronal adenosine A1 receptors, thereby enhancing ATP production [13]. The KD has recently been used in neurological disorders, including Alzheimer’s and Parkinson’s disease, as an alternative brain fuel that enhances mitochondrial function and biogenesis [14, 15]. Its effects have also been studied in genetically determined metabolic disorders. KD is the recommended therapy for pyruvate dehydrogenase complex (PDHc) deficiency and glucose transporter type 1 deficiency syndrome (GLUT1‐DS), as it targets the underlying metabolic defect, influencing mitochondrial bioenergetics, ROS/redox balance, and dynamics [16].

Regarding the pathophysiological mechanisms underlying the beneficial effects of the KD on mitochondrial function, the available evidence derives primarily from animal models and in vitro studies [17]. The ability of ketone bodies to stimulate mitogenesis provides a plausible explanation for the effectiveness of the KD in reducing lactate levels and improving clinical conditions in patients with FBXL4‐related mitochondrial dysfunction, which is characterized by a substantial upregulation of mitophagy.

The observation of diffuse hyperechogenic periventricular white matter in our patient falls within the recognized MRI spectrum of FBXL4‐related disorders, representing a form of white‐matter abnormality. This finding is consistent with previously described MRI features—such as leukodystrophic changes and delayed myelination—which further supports the notion that brain involvement in FBXL4 deficiency may already be present during the antenatal period [18].

The echocardiographic findings in our patient also appear consistent with those previously reported in individuals affected by MTDPS13 [19]. In addition, the presence of pigmentary changes in the macula has been described only once before [20].

In conclusion, our case highlights the feasibility of implementing a ketogenic regimen in a preterm infant, with a gradual transition from parenteral to enteral and ultimately oral nutrition, and demonstrates that this approach can be safe and practicable when performed under close monitoring. Dietary intervention with a KD is increasingly employed for the symptomatic management of patients with mitochondrial diseases, given its cost‐effectiveness and ready availability. Early initiation of a ketogenic regimen may help control metabolic symptoms and improve survival, including in infants with severe mutations of MTDPS13.

## Funding

No funding was received for this manuscript.

## Conflicts of Interest

The authors declare no conflicts of interest.

## Data Availability

The data that support the findings of this study are available from the corresponding author upon reasonable request.

## References

[1] Wallace D. C. , A Mitochondrial Paradigm of Metabolic and Degenerative Diseases, Aging, and Cancer: A Dawn for Evolutionary Medicine, Annual Review of Genetics. (2005) 39, no. 1, 359–407, 10.1146/annurev.genet.39.110304.095751, 2-s2.0-23844558266. PMC2821041 16285865

[2] Schon E. , DiMauro S. , and Hirano M. , Human Mitochondrial DNA: Roles of Inherited and Somatic Mutations, Nature Reviews Genetics. (2012) 13, no. 12, 878–890, 10.1038/nrg3275, 2-s2.0-84869397441. PMC3959762 23154810

[3] Sabouny R. and Shutt T. E. , The Role of Mitochondrial Dynamics in mtDNA Maintenance, Journal of Cell Science. (2021) 134, no. 24, 10.1242/jcs.258944. 34910819

[4] El-Hattab A. W. and Scaglia F. , Mitochondrial DNA Depletion Syndromes: Review and Updates of Genetic Basis, Manifestations, and Therapeutic Options, Neurotherapeutics. (2013) 10, no. 2, 186–198, 10.1007/s13311-013-0177-6, 2-s2.0-84876180436. 23385875 PMC3625391

[5] Spinazzola A. , Mitochondrial DNA Mutations and Depletion in Pediatric Medicine, Seminars in Fetal and Neonatal Medicine. (2011) 16, no. 4, 190–1966, 10.1016/j.siny.2011.04.011, 2-s2.0-79959892261. 21652274

[6] Bonnen P. E. , Yarham J. W. , Besse A. et al., Mutations in FBXL4 Cause Mitochondrial Encephalopathy and a Disorder of Mitochondrial DNA Maintenance, American Journal of Human Genetics. (2013) 93, no. 3, 471–481, 10.1016/j.ajhg.2013.07.017, 2-s2.0-84883810020. 23993193 PMC3769921

[7] Xiaowu G. , Ghezzi D. , Johnson M. A. et al., Mutations in FBXL4, Encoding a Mitochondrial Protein, Cause Early-Onset Mitochondrial Encephalomyopathy, American Journal of Human Genetics. (2013) 93, no. 3, 482–495, 10.1016/j.ajhg.2013.07.016, 2-s2.0-84883780647. 23993194 PMC3769923

[8] Joosten K. and Vermeulen M. , Principles of Feeding the Preterm Infant, Clinical Nutrition ESPEN. (2024) 59, 320–327, 10.1016/j.clnesp.2023.12.016. 38220393

[9] Embleton N. D. , Moltu J. , Lapillonne A. et al., Enteral Nutrition in Preterm Infants (2022): A Position Paper From the ESPGHAN Committee on Nutrition and Invited Experts, Journal of Pediatric Gastroenterology and Nutrition. (2023) 76, 248–268, 10.1097/MPG.0000000000003642. 36705703

[10] Simpson C. , Schanler R. J. , and Lau C. , Early Introduction of Oral Feeding in Preterm Infants, Pediatrics. (2002) 110, no. 3, 517–522, 10.1542/peds.110.3.517, 2-s2.0-0036725971. 12205253

[11] İnci A. , Aktaş E. , Cengiz Ergin F. B. et al., The First Case With FBXL4 Mutation Successfully Treated With a Parenteral Ketogenic Diet for Lactic Acidosis, Journal of Parenteral and Enteral Nutrition. (2021) 45, no. 8, 1788–1792, 10.1002/jpen.2121. 33882172

[12] Köse E. , Köse M. , Edizer S. et al., Different Clinical Presentation in a Patient With Two Novel Pathogenic Variants of the FBXL4 Gene, Turkish Journal of Pediatrics. (2020) 62, no. 4, 652–656, 10.24953/turkjped.2020.04.016. 32779419

[13] D’Andrea M. I. , Romão T. T. , Pires do Prado H. J. , Krüger L. T. , Pires M. E. P. , and da Conceição P. O. , Ketogenic Diet and Epilepsy: What We Know so Far, Frontiers in Neuroscience. (2019) 13, 10.3389/fnins.2019.00005, 2-s2.0-85065502987. PMC6361831 30760973

[14] Pietrzak D. , Kasperek K. , Rękawek P. , and Piątkowska-Chmiel I. , The Therapeutic Role of Ketogenic Diet in Neurological Disorders, Nutrients. (2022) 14, no. 9, 10.3390/nu14091952. PMC9102882 35565918

[15] Zweers H. , van Wegberg A. M. J. , Janssen M. C. H. , and Wortmann S. B. , Ketogenic Diet for Mitochondrial Disease: A Systematic Review on Efficacy and Safety, Orphanet Journal of Rare Diseases. (2021) 16, 10.1186/s13023-021-01927-w. PMC8254320 34217336

[16] Tummolo A. , Carella R. , De Giovanni D. et al., Micronutrient Deficiency in Inherited Metabolic Disorders Requiring Diet Regimen: A Brief Critical Review, International Journal of Molceular Sciences. (2023) 24, no. 23, 10.3390/ijms242317024. PMC10707160 38069347

[17] Harun-Or-Rashid M. , Pappenhagen N. , Palmer P. G. et al., Structural and Functional Rescue of Chronic Metabolically Stressed Optic Nerves Through Respiration, Journal of Neuroscience. (2018) 38, no. 22, 5122–5139, 10.1523/JNEUROSCI.3652-17.2018, 2-s2.0-85050811316. 29760184 PMC5977447

[18] Saini N. , Vijayasree V. , Nandury E. C. , Dalal A. , and Aggarwal S. , Prenatal Phenotype of FBXL4-Associated Encephalomyopathic Mitochondrial DNA Depletion Syndrome-13, Prenatal Diagnosis. (2022) 42, no. 13, 1682–1685, 10.1002/pd.6272. 36411461

[19] Nardi N. , Proulx F. , Brunel-Guiton C. et al., Fulminant Necrotizing Enterocolitis and Multiple Organ Dysfunction in a Toddler With Mitochondrial DNA Depletion Syndrome-13, Journal of Pediatric Intensive Care. (2020) 9, no. 01, 54–59, 10.1055/s-0039-1697620. 31984159 PMC6978174

[20] Kahraman A. B. , Çelik H. , Bagci Z. , Sezer A. , and Kılıç M. , FBXL4-Related Encephalomyopathic Mitochondrial DNA Depletion Syndrome: A Rare Cause of Hyperammonemia, Molecular Genetics and Metabolism Reports. (2025) 43, 10.1016/j.ymgmr.2025.101206. PMC11952866 40161922

